# A healthy lifestyle mitigates the risk of heart disease related to type 2 diabetes: a prospective nested case–control study in a nationwide Swedish twin cohort

**DOI:** 10.1007/s00125-020-05324-z

**Published:** 2020-11-10

**Authors:** Rongrong Yang, Hui Xu, Nancy L. Pedersen, Xuerui Li, Jing Yu, Cuiping Bao, Xiuying Qi, Weili Xu

**Affiliations:** 1 Public Health Science and Engineering College, Tianjin University of Traditional Chinese Medicine, Tianjin, China; 2 Department of Epidemiology and Biostatistics, School of Public Health, Tianjin Medical University, Tianjin, China; 3 Big Data and Engineering Research Center, Beijing Children’s Hospital, Capital Medical University, National Center for Children’s Health, Beijing, China; 4 Department of Medical Epidemiology and Biostatistics, Karolinska Institutet, Stockholm, Sweden; 5 Department of Psychology, University of Southern California, Los Angeles, CA USA; 6 Department of Physiology and Pathophysiology, School of Basic Medicine, Tianjin Medical University, Tianjin, China; 7 Department of Radiology, Tianjin Union Medical Centre, Tianjin, China; 8 Aging Research Center, Department of Neurobiology, Health Care Sciences and Society, Karolinska Institutet and Stockholm University, Stockholm, Sweden

**Keywords:** Heart disease, Lifestyle, Prospective nested case–control study in twins, The Swedish Twin Registry, Type 2 diabetes

## Abstract

**Aims/hypothesis:**

We aimed to examine the association between type 2 diabetes and major subtypes of heart disease, to assess the role of genetic and early-life familial environmental factors in this association and to explore whether and to what extent a healthy lifestyle mitigates the risk of heart disease related to type 2 diabetes.

**Methods:**

In this prospective nested case–control study based on the Swedish Twin Registry, 41,463 twin individuals who were aged ≥40 and heart disease-free were followed up for 16 years (from 1998 to 2014) to detect incident heart disease. Type 2 diabetes was ascertained from self-report, the National Patient Registry and glucose-lowering medication use. Heart disease diagnosis (including coronary heart disease, cardiac arrhythmias and heart failure) and onset age were identified from the National Patient Registry. Healthy lifestyle-related factors consisted of being a non-smoker, no/mild alcohol consumption, regular physical activity and being non-overweight. Participants were divided into three groups according to the number of lifestyle-related factors: (1) unfavourable (participants who had no or only one healthy lifestyle factor); (2) intermediate (any two or three); and (3) favourable (four). Generalised estimating equation models for unmatched case–control design and conditional logistic regression for co-twin control design were used in data analyses.

**Results:**

Of all participants, 2304 (5.5%) had type 2 diabetes at baseline. During the observation period, 9262 (22.3%) had any incident heart disease. In unmatched case–control analyses and co-twin control analyses, the multi-adjusted OR and 95% CI of heart disease related to type 2 diabetes was 4.36 (3.95, 4.81) and 4.89 (3.88, 6.16), respectively. The difference in ORs from unmatched case–control analyses vs co-twin control analyses was statistically significant (OR 1.57; 95% CI 1.42, 1.73; *p* < 0.001). In stratified analyses by type 2 diabetes, compared with an unfavourable lifestyle, an intermediate lifestyle or a favourable lifestyle was associated with a significant 32% (OR 0.68; 95% CI 0.49, 0.93) or 56% (OR 0.44; 95% CI 0.30, 0.63) decrease in heart disease risk among patients with type 2 diabetes, respectively. There were significant additive and multiplicative interactions between lifestyle and type 2 diabetes on heart disease.

**Conclusions/interpretation:**

Type 2 diabetes is associated with more than fourfold increased risk of heart disease. The association still remains statistically significant, even after fully controlling for genetic and early-life familial environmental factors. However, greater adherence to a healthy lifestyle may significantly mitigate the risk of heart disease related to type 2 diabetes.

**Figure:**
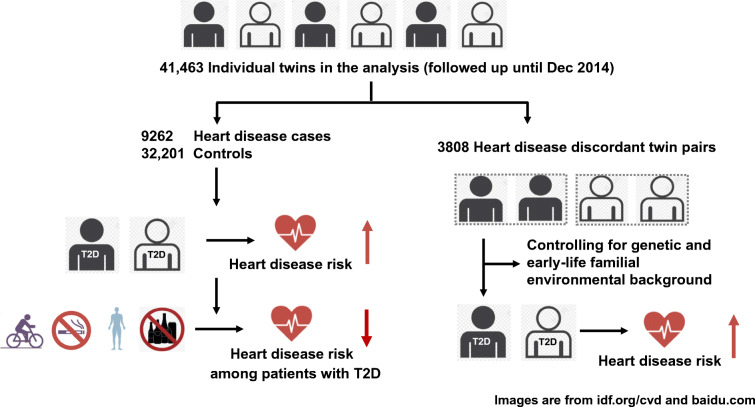
Graphical abstract

**Supplementary Information:**

The online version contains supplementary material available at 10.1007/s00125-020-05324-z.

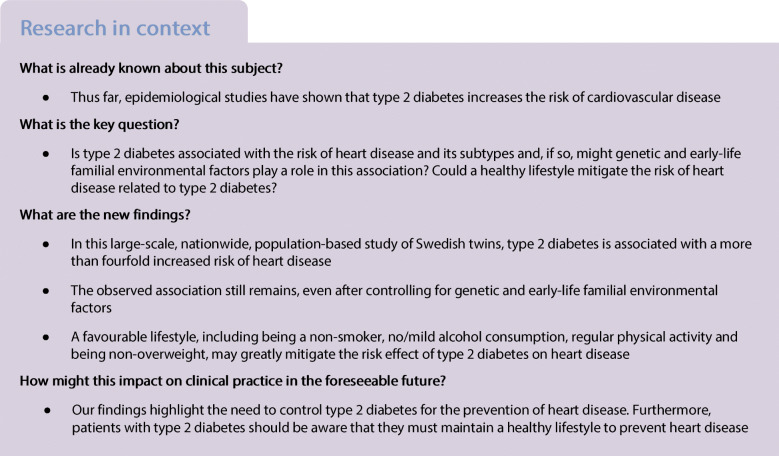



## Introduction

Worldwide, diabetes affected 451 million people (8.4% of the world’s population) in 2017, and this number might dramatically rise to 693 million (9.9%) by 2045 [1]. Patients with type 2 diabetes are at increased risk of several chronic diseases and associated clinical complications, such as heart disease [2], which, in turn, is associated with cerebral vascular disease, dementia, disability and premature mortality [3].

Coronary heart disease, heart failure and cardiac arrhythmias are the common types of heart disease [3]. Thus far, population-based longitudinal studies have consistently shown that type 2 diabetes is associated with the risk of total CVD, mainly including coronary heart disease and stroke [2, 4–7]. However, the associations between type 2 diabetes and certain subtypes of heart disease independently remain unclear. Several cohort studies examined the relationship between type 2 diabetes and atrial fibrillation and flutter, and showed inconsistent results [8–12]. Discrepancies in previous findings can be attributed to the different study populations, follow-up times and sample size, and lack of consideration of possible confounders.

Although type 2 diabetes may be linked to heart disease through several biologically plausible pathways, our understanding of the mechanisms for such an association is still limited. Both type 2 diabetes and heart disease are complex genetic and lifestyle-related disorders [3]. Genetic and early-life familial environmental factors may contribute to the development of type 2 diabetes [13] and heart disease [14]. However, their role in the association between type 2 diabetes and heart disease is uncertain. Twins are generally reared together and share genetic background. Thus, twin studies provide the possibility to assess whether genetic and/or early familial environmental factors play a role in a given association [15]. In addition, previous studies have suggested that an individual healthy lifestyle factor (such as maintaining a normal weight, being a non-smoker, non-heavy drinking or regular exercise) was associated with lower risk of both type 2 diabetes and CVD in the general population [3, 16]. Currently, accumulating evidence has shown that adopting an overall and combined healthy lifestyle can be a more effective prevention strategy for patients with type 2 diabetes to reduce the risk of cardiovascular complications (such as cause-specific mortality rate) [17, 18]. However, the question remains whether and to what extent a combined healthy lifestyle may counteract the risk of heart disease associated with type 2 diabetes.

In the current study, we sought to: (1) examine the association between type 2 diabetes and the risk of heart disease including its major subtypes; (2) explore whether genetic and early-life familial environmental factors play a role in this association; and (3) investigate whether and to what extent a healthy lifestyle could mitigate the risk of heart disease related to type 2 diabetes.

## Methods

### Study population

**Fig. 1:**
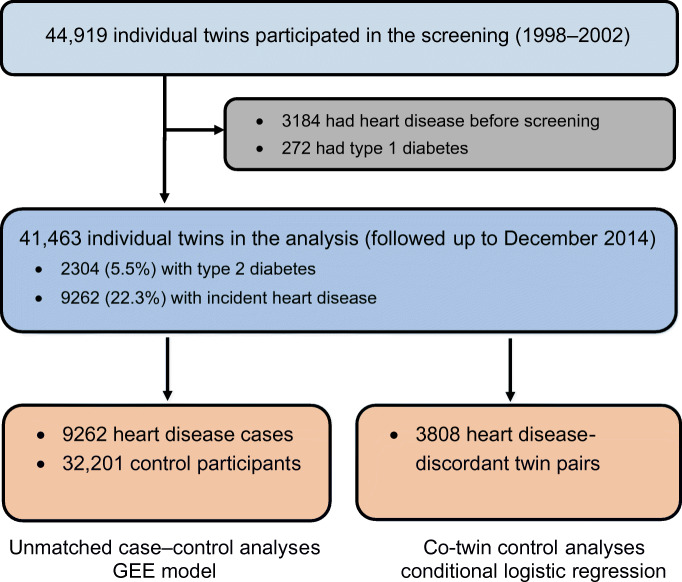
Flowchart of the study population

This prospective, nested case–control study included twins from the nationwide Swedish Twin Registry (STR), which was started in the 1960s [19]. In 1998–2002, all living twins in the registry who were born in 1958 or earlier were invited to participate in the Screening Across the Lifespan Twin study (SALT), a full-scale screening that gathered data on an extended set of variables via computer-assisted telephone interviews. Out of 44,919 twin individuals eligible for the telephone interview, we excluded 3184 with heart disease before screening and 272 with type 1 diabetes, resulting in 41,463 individuals with data for the current analyses (Fig. 1).

### Data collection

Information on age, sex, educational attainment, marital status and zygosity was obtained from the SALT survey [19]. All twins were categorised as monozygotic, dizygotic or of undetermined zygosity. Education was defined as the maximum years of formal schooling attained, and dichotomised into <8 vs ≥8 years. Marital status was defined as married/cohabiting vs single (including divorced and widows/widowers).

Information on history of type 2 diabetes and heart disease was derived from the National Patient Registry (NPR), which covers all inpatient diagnoses in Sweden from the 1960s to the end of 2014, and outpatient (specialist clinic) diagnoses since 2001. Each medical record in the NPR included up to eight discharge diagnoses according to the ICD. The seventh revision (ICD-7) was used up to 1968, the eighth revision (ICD-8) from 1969 to 1986, the ninth revision (ICD-9) from 1987 to 1996 and the tenth revision (ICD-10) since 1997.

All participants provided informed consent. The data collection procedures were approved by the Regional Ethics Committee at Karolinska Institutet, Stockholm, Sweden, and by the Institutional Review Board of the University of Southern California, USA.

### Ascertainment of type 2 diabetes

Type 2 diabetes was ascertained based on self- and informant-reported history of diabetes, glucose-lowering medication use or the NPR (ICD-7 code 260; ICD-8 and -9 code 250; and ICD-10 codes E10–E14). The age at type 2 diabetes onset was estimated according to the earliest recorded date of type 2 diabetes in the NPR or the date of type 2 diabetes onset available in SALT.

### Assessment of heart disease

Information on heart disease diagnoses (ICD-7 codes 420, 433 and 434; ICD-8 and -9 codes 410–414, 427 and 428; and ICD-10 codes I20–I25 and I48–I50) was obtained from the NPR. According to the ICD codes, the major subtypes of heart disease included: (1) coronary heart disease: angina pectoris, acute myocardial infarction, chronic ischaemic heart disease and other coronary heart disease (such as coronary thrombosis and Dressler’s syndrome); (2) cardiac arrhythmias: atrial fibrillation and flutter, and other cardiac arrhythmias (such as ventricular fibrillation and flutter, atrial premature depolarisation and junctional premature depolarisation); and (3) heart failure: congestive heart failure, left ventricular failure and unspecified heart failure. The age of heart disease onset was estimated as the earliest date that a heart disease diagnosis was recorded in the NPR.

### Assessment of lifestyle-related factors

Information on smoking status, alcohol consumption, physical activity and BMI was obtained from the SALT survey. Smoking status was dichotomised as never vs ever being a smoker. Data on alcohol consumption were collected by a question on drinking habits, ‘Think about your use of alcohol over your entire life. Has there ever been a period in your life when you drank too much?’, with two response options: (1) ‘no’; and (2) ‘yes’. We defined ‘no’ as ‘no/mild drinking’ and ‘yes’ as ‘heavy drinking’. Data on physical activity were collected by a question on average exercise, with seven response options: (1) ‘almost never’; (2) ‘much less than average’; (3) ‘less than average’; (4) ‘average’; (5) ‘more than average’; (6) ‘much more than average’; and (7) ‘maximum’ [20]. For the analyses, we combined these categories into two groups and defined ‘low’ as exercise ‘almost never’ to ‘much less than average’, and ‘regular’ physical activity as ‘less than average’ to ‘maximum’. BMI was calculated as weight (kg) divided by height squared (m^2^), and was categorised as non-overweight (BMI <25) and overweight (BMI ≥25).

In the current study, we considered four healthy lifestyle-related factors: being a non-smoker, no/mild alcohol consumption, regular physical activity and being non-overweight. Participants were divided into three groups according to the number of lifestyle-related factors: (1) unfavourable: participants who had no or only one healthy lifestyle factor; (2) intermediate: those who had any two or three healthy lifestyle factors; and (3) favourable: those who had four healthy lifestyle factors.

### Statistical analysis

The characteristics of participants in different groups were compared using *χ*^2^ tests, *t* test and Mann–Whitney test. Generalised estimating equation (GEE) models were used to analyse the unmatched case–control data while controlling for the clustering of twins within a pair. To examine the associations between type 2 diabetes and risk of heart disease independently, we looked at the first onset of one specific subtype of heart disease with no others. Data for the co-twin control study were analysed by using conditional logistic regression, in which twin pairs were discordant for outcome; thus, cases and control participants were comparable with respect to early-life familial environmental factors (such as shared childhood socioeconomic status and adolescent environment) and genetic background (monozygotic twins shared 100% of their genetic background and dizygotic twins shared only 50%) [15]. In both GEE and conditional logistic regression, the ORs and 95% CIs were estimated for the association between type 2 diabetes and heart disease.

Logistic regression was used to test the difference in ORs from GEE models and conditional logistic regression by examining the difference between the proportions of type 2 diabetes in unmatched control participants and in co-twin control participants [21–24]. Absence of a statistically significant difference in ORs from the GEE and conditional logistic regression analyses suggests that genetic and early-life familial environmental factors might not account for the observed associations. In contrast, a statistically significant difference in ORs from the GEE and conditional logistic regression analyses indicates that genetic and/or shared environmental factors likely play a role in the observed associations [15, 21–25].

The combined effect of the type 2 diabetes and lifestyle on heart disease risk was assessed by creating dummy variables based on the joint exposures to both factors. The presence of an additive interaction was examined by estimating the relative excess risk due to interaction (RERI), the attributable proportion (AP) and the synergy index (SI). Additionally, we examined multiplicative interaction by incorporating the two variables and their cross-product term in the same model.

Age, sex, education, BMI, smoking, alcohol consumption, marital status and physical activity were considered as potential confounders in the type 2 diabetes–heart disease association. Missing values on education (*n* = 1217), smoking (*n* = 1167), alcohol consumption (*n* = 1261), BMI (*n* = 1918), marital status (*n* = 755) and physical activity (*n* = 5938) were imputed by chained equation to obtain valid statistical inferences with five completed datasets generated. All statistical analyses were performed using SAS statistical software version 9.4 (SAS Institute, Cary, NC, USA) and IBM SPSS Statistics 24.0 (IBM Corp, New York, NY, USA).

## Results

### Characteristics of the study population

Among all participants, 18,838 (45.4%) were men and 22,625 (54.6%) were women (*χ*^2^ = 30.95, *p* < 0.001). In total, 2304 (5.5%) had type 2 diabetes. Compared with type 2 diabetes-free participants, those with type 2 diabetes were more likely to be older, male, single and non-smokers; to engage in low levels of physical activity; and to have lower educational attainment and higher BMI (Table 1).

**Table 1:** Baseline characteristics of the study participants by type 2 diabetes status (*N* = 41,463)

Characteristic	T2D-free *n* = 39,159	T2D *n* = 2304	*p* value
Age (years), mean ± SD	58.1 ± 10.6	64.7 ± 10.5	<0.001
Male sex, *n* (%)	17,662 (45.1)	1176 (51.0)	<0.001
Education, *n* (%)			<0.001
<8 years	12,510 (32.0)	1091 (47.4)	
≥8 years	26,649 (68.0)	1213 (52.6)	
Marital status, *n* (%)			<0.001
Married/cohabiting	28,626 (73.1)	1487 (64.5)	
Single	10,533 (26.9)	817 (35.5)	
Zygosity, *n* (%)			0.879
Monozygotic	7759 (19.8)	458 (19.9)	
Dizygotic	26,233 (67.0)	1534 (66.6)	
Undetermined	5167 (13.2)	312 (13.5)	
BMI, mean ± SD	24.8 ± 3.4	26.9 ± 4.1	<0.001
<25 (Non-overweight)	21,880 (55.9)	768 (33.3)	<0.001
≥25 (Overweight)	17,279 (44.1)	1536 (66.7)	
Smoking status, *n* (%)			0.024
Never	19,058 (48.7)	1177 (51.1)	
Ever a smoker	20,101 (51.3)	1127 (48.9)	
Alcohol consumption, *n* (%)			0.247
No/mild drinking	36,204 (92.5)	2115 (91.8)	
Heavy drinking	2955 (7.5)	189 (8.2)	
Physical activity, *n* (%)			0.007
Low	3905 (10.0)	270 (11.7)	
Regular	35,254 (90.0)	2034 (88.3)	

Data are presented as mean ± SD or number (proportion, %)

T2D, type 2 diabetes

### Association between type 2 diabetes and heart disease in unmatched case–control analyses

During 1998–2014, 9262 (22.3%) participants had incident heart disease. Compared with type 2 diabetes-free participants, the multi-adjusted OR for any heart disease associated with type 2 diabetes was 4.36 (95% CI 3.95, 4.81); for angina pectoris, OR 4.23 (95% CI 3.62, 4.94); for acute myocardial infarction, OR 4.93 (95% CI 4.25, 5.72); for chronic ischaemic heart disease, OR 5.14 (95% CI 3.82, 6.91); for atrial fibrillation and flutter, OR 3.14 (95% CI 2.71, 3.64); for congestive heart failure, OR 5.76 (95% CI 3.96, 8.38); and for left ventricular failure, OR 4.45 (95% CI 2.65, 7.49) (Table 2).

**Table 2:** ORs and 95% CIs of different forms of heart disease related to type 2 diabetes from GEE models (type 2 diabetes-free as the reference)

Heart disease	No. of cases	OR (95% CI)^a^	OR (95% CI)^b^
All types	9262	4.71 (4.27, 5.19)	4.36 (3.95, 4.81)
Coronary heart disease	4403	5.14 (4.58, 5.78)	4.83 (4.30, 5.43)
Angina pectoris	1936	4.53 (3.88, 5.29)	4.23 (3.62, 4.94)
Acute myocardial infarction	2089	5.20 (4.49, 6.03)	4.93 (4.25, 5.72)
Chronic ischaemic heart disease	362	5.49 (4.09, 7.37)	5.14 (3.82, 6.91)
Other coronary heart disease	16	15.08 (4.85, 46.86)	15.39 (4.69, 50.49)
Cardiac arrhythmias	3471	3.35 (2.92, 3.84)	3.14 (2.74, 3.60)
Atrial fibrillation and flutter	2835	3.40 (2.94, 3.94)	3.14 (2.71, 3.64)
Other cardiac arrhythmias	636	3.03 (2.28, 4.03)	3.04 (2.28, 4.04)
Heart failure	1388	5.31 (4.45, 6.34)	4.89 (4.09, 5.84)
Congestive heart failure	181	6.29 (4.30, 9.21)	5.76 (3.96, 8.38)
Left ventricular failure	110	4.92 (2.91, 8.29)	4.45 (2.65, 7.49)
Unspecified heart failure	1097	4.86 (4.01, 5.90)	4.52 (3.72, 5.49)

^a^Adjusted for age, sex and education

^b^Additionally adjusted for marital status, BMI, smoking, alcohol consumption and physical activity

### Association between type 2 diabetes and heart disease in co-twin control analyses

Compared with the OR in GEE models, the association between type 2 diabetes and heart disease became stronger (OR 4.89; 95% CI 3.88, 6.16) in the co-twin control analyses (including all twin pairs). The difference in ORs from the GEE models based on unmatched case–control analyses vs co-twin control analyses in all twin pairs was statistically significant (OR 1.57; 95% CI 1.42, 1.73; *p* < 0.001). In addition, the multi-adjusted OR (95% CI) of heart disease associated with type 2 diabetes was 4.07 (3.15, 5.27) in dizygotic twins and 10.83 (4.67, 25.10) in monozygotic twins. These results suggest that type 2 diabetes is still associated with heart disease, even after fully controlling for genetic and early-life familial environmental factors (Table 3).

**Table 3:** ORs and 95% CIs for the association between type 2 diabetes and heart disease in co-twin control analyses using conditional logistic regression

Co-twin without heart disease	Co-twin with heart disease					
	All types^a^		Dizygotic only		Monozygotic only	
	T2D-free	T2D	T2D-free	T2D	T2D-free	T2D
T2D-free	3193	464	2278	337	588	65
T2D	90	61	76	36	6	17
Basic-adjusted OR (95% CI)^b^	5.07 (4.04, 6.38)		4.32 (3.35, 5.56)		10.88 (4.71, 25.11)	
Multi-adjusted OR (95% CI)^c^	4.89 (3.88, 6.16)		4.07 (3.15, 5.27)		10.83 (4.67, 25.10)	

^a^Including dizygotic twins, monozygotic twins and twins of undetermined zygosity. The 3808 heart disease-discordant pairs were divided into four groups with respect to exposure (T2D) status. In 3193 twin pairs, both were T2D-free. In 61 twin pairs, both had T2D. In 464 twin pairs, the healthy (heart disease-free) co-twin was T2D-free and the diseased twin had T2D. In 90 twin pairs, the diseased co-twin was T2D-free and the healthy twin had T2D

^b^Adjusted for sex and education

^c^Adjusted for sex, education, marital status, BMI, smoking, alcohol consumption and physical activity

T2D, type 2 diabetes

### Association between lifestyle-related factors and heart disease

In multi-adjusted GEE models, being a non-smoker, regular physical activity, no/mild drinking and being non-overweight were associated with a decreased risk of heart disease. In further analysis, an intermediate lifestyle and a favourable lifestyle were significantly associated with a lower risk of heart disease (Table 4).

**Table 4:** ORs and 95% CIs of heart disease in relation to BMI, smoking status, alcohol consumption and physical activity from GEE models

Lifestyle factor	No. of cases	Heart disease OR (95% CI)^a^	Heart disease OR (95% CI)^b^
BMI			
≥25 (overweight)	4955	Reference	Reference
<25 (non-overweight)	4307	0.71 (0.68, 0.75)	0.77 (0.74, 0.81)
Smoking			
Yes	4697	Reference	Reference
No	4565	0.86 (0.81, 0.90)	0.86 (0.82, 0.91)
Alcohol consumption			
Heavy drinking	710	Reference	Reference
No/mild drinking	8552	0.83 (0.76, 0.91)	0.89 (0.81, 0.98)
Physical activity			
Low	1032	Reference	Reference
Regular	8230	0.78 (0.72, 0.84)	0.82 (0.76, 0.89)
Lifestyle			
Unfavourable	693	Reference	Reference
Intermediate	6638	0.70 (0.64, 0.77)	0.73 (0.66, 0.81)
Favourable	1931	0.49 (0.44, 0.55)	0.54 (0.48, 0.60)

^a^Adjusted for age, sex and education

^b^Adjusted for age, sex, education, marital status and type 2 diabetes, as well as BMI, smoking, alcohol consumption and physical activity, if applicable

**Fig. 2:**
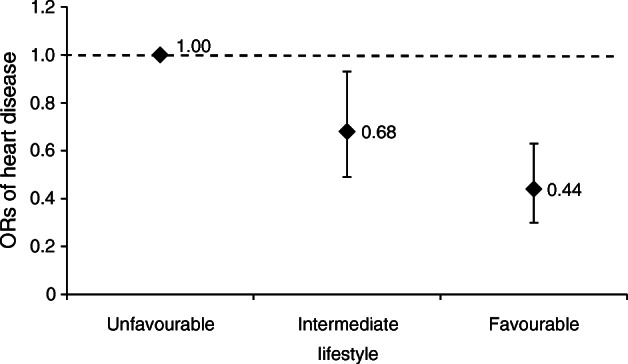
Multi-adjusted ORs (95% CIs) of heart disease in relation to lifestyle among patients with type 2 diabetes from GEE models (adjusted for age, sex, education and marital status)

In stratified analyses by type 2 diabetes, compared with an unfavourable lifestyle, an intermediate lifestyle or a favourable lifestyle was associated with a significant 32% (OR 0.68; 95% CI 0.49, 0.93) or 56% (OR 0.44; 95% CI 0.30, 0.63) decrease in heart disease risk among patients with type 2 diabetes, respectively (Fig. 2 and electronic supplementary material [ESM] Table 1).

### Joint effect of type 2 diabetes and lifestyle-related factors on heart disease risk

In joint effect analyses, there was a significant additive interaction between type 2 diabetes and lifestyle on heart disease risk (RERI 3.507; 95% CI 0.929, 6.084; AP 0.414; 95% CI 0.231, 0.597; SI 1.885; 95% CI 1.318, 2.696) (ESM Table 2). The multi-adjusted OR for type 2 diabetes multiplied by unfavourable lifestyle was 1.30 (95% CI 1.07, 1.57; *p* = 0.008) for heart disease.

### Supplementary analysis

Considering possible sex differences in heart disease development, we performed stratified analysis, and the associations between type 2 diabetes and heart disease risk did not vary by sex (ESM Table 3). The results were not much altered compared with those from initial analyses when we repeated analyses: (1) with additional adjustment for survival status (ESM Table 4); and (2) excluding missing values for covariates (ESM Table 5).

## Discussion

In this large-scale, nationwide, genetically informative sample of Swedish twins, we found that type 2 diabetes was independently associated with increased risk of heart disease and its major types, specifically angina pectoris, acute myocardial infarction, chronic ischaemic heart disease, atrial fibrillation and flutter, and heart failure. The association remained significant, even after controlling for genetic and early-life familial environmental factors. However, a healthy lifestyle might significantly mitigate the risk of heart disease related to type 2 diabetes compared with an unfavourable lifestyle.

In recent decades, many epidemiologic studies have shown that type 2 diabetes is associated with a two- to sixfold increased risk of total CVD and coronary heart disease [2, 4–6]. However, the association between type 2 diabetes and atrial fibrillation has been addressed in a number of epidemiologic studies with inconclusive results. Some studies showed an increased risk of atrial fibrillation among people with type 2 diabetes [8, 9, 12], but others indicated no clear association [10, 11]. In a recent meta-analysis of 32 cohort studies, type 2 diabetes was associated with a modest 30% increased atrial fibrillation risk [26]. In the present study, we found that type 2 diabetes conferred a more than fourfold greater risk of coronary heart disease and a doubled atrial fibrillation risk. Several studies have shown that type 2 diabetes is positively associated with heart failure, but in most of these the influences of other subtypes of heart disease were not taken into account [2, 27, 28]. As the onset and progression of angina pectoris, acute myocardial infarction and atrial fibrillation may also contribute to heart failure, we looked at the first onset of heart failure with no previous coronary heart disease and cardiac arrhythmias and found that the higher risk of heart failure with type 2 diabetes was independent of other specific subtypes of heart disease.

Accumulating evidence has shown that molecular defects, intrauterine environment and socioeconomic factors are associated with the development of type 2 diabetes, and also contribute to an increased risk of heart disease [13, 29]. Twins are generally raised together and share the same genetic background as well as intrauterine, childhood and adolescent environments. Twin studies provide us with an opportunity to investigate whether the association between type 2 diabetes and heart disease is potentially confounded by genetic and/or early-life familial environmental backgrounds. In the present study, results of co-twin control analyses implicate that type 2 diabetes is still associated with an increased risk of heart disease, even after fully controlling for genetic and early-life familial environmental backgrounds.

Thus far, previous studies have mainly focused on the combined effect of an overall healthy lifestyle and type 2 diabetes on mortality or total CVD (including coronary heart disease, stroke and peripheral vascular disease) risk, but data specific for only heart disease risk are limited. One population-based prospective cohort study of Chinese patients with type 2 diabetes showed that active smoking, physical inactivity, alcohol drinking and high carbohydrate intake increased the risk of all-cause mortality and CVD mortality after a mean follow-up of 4.02 years of follow-up [17]. Another prospective study including 11,527 participants with type 2 diabetes suggested that an overall healthy lifestyle (diet, smoking status, alcohol consumption and physical activity) was associated with substantially lower risks of CVD incidence (including stroke and coronary heart disease) and CVD mortality during a mean follow-up of 13.3 years of follow-up [18]. In contrast, at a median follow-up of almost 10 years, a multicentre randomised clinical trial found that an intensive lifestyle intervention (diet modification and increased physical activity) could produce improvements in CVD risk factors (such as blood pressure and high-density lipoprotein cholesterol levels) in individuals with type 2 diabetes, but not reduce CVD events (including stroke and coronary heart disease) [30]. The discrepancy in findings might reflect differences in follow-up times, lifestyle factors and definitions of outcome. To the best of our knowledge, the current study is the first to provide evidence that a healthy lifestyle consisting of being a non-smoker, no/mild alcohol consumption, regular physical activity and being non-overweight may greatly attenuate the risk of heart disease in type 2 diabetes. In the current study, patients with type 2 diabetes who reported maintaining not only a favourable (four healthy lifestyle factors) but also an intermediate lifestyle (any two or three healthy lifestyle factors) had a significantly lower heart disease risk than those with an unfavourable lifestyle (no or only one healthy lifestyle factor).

The mechanisms responsible for the increased heart disease morbidity attributable to type 2 diabetes are multifactorial and incompletely understood. An important role of metabolic disturbances, such as long-term hyperglycaemia, insulin resistance and dyslipidaemia, has been hypothesised [31, 32]. Accelerated atherosclerosis and thrombosis in patients with type 2 diabetes principally result from inflammation, reactive oxygen species and endothelial dysfunction combined with coagulation, platelet abnormalities and impaired fibrinolysis [33]. Type 2 diabetes leads to autonomic dysfunction and structural remodelling of the left atrium in the form of atrial dilatation and interstitial fibrosis, which might contribute to life-threatening arrhythmias [26]. In addition, how a favourable lifestyle mitigates the risk of heart disease among participants with or without type 2 diabetes may be explained by multiple possible mechanisms—an overall healthy lifestyle can improve glycaemic control, insulin sensitivity, blood pressure, platelet function, lipid profile and body composition [16, 17, 34].

There are several strengths and limitations in the current study. First, the large, nationwide, population-based twin cohort provided us with a unique opportunity to further examine the effect of type 2 diabetes on heart disease risk while controlling for some unmeasured confounders such as genetic and early-life familial environmental factors. Second, we used GEE modelling, which is more appropriate than logistic regression models in case–control design, since it accounts for the clustering of twins within a pair. Nonetheless, the limitations in our study need to be pointed out. First, blood glucose level was not available in the STR or SALT. Consequently, given the higher prevalence of undiagnosed type 2 diabetes in elderly people [35], subjects with undiagnosed type 2 diabetes might have been misclassified as type 2 diabetes-free, which might have led to an underestimation of the observed associations. Second, type 2 diabetes and heart disease were associated with mortality risk, which may contribute to under- or over-estimation of the observed associations. In the current study, we repeated the analyses with an additional adjustment for survival status, and the results were not substantially altered. Third, because information on lifestyle factors was obtained at baseline, it is difficult to capture potential variations in lifestyle factors during follow-up, which would result in underestimation for the effect. Fourth, although some lifestyle-related factors such as smoking, alcohol consumption and physical activity were taken into account, information on diet, sleep duration and other lifestyle-related factors was not available. Finally, information bias might have occurred due to self-reported information on lifestyle-related factors. This might have caused non-differential misclassification leading to underestimation for the observed association.

### Conclusions

In conclusion, our study provides further evidence that type 2 diabetes is associated with about fourfold greater risk of heart disease, including coronary heart disease, cardiac arrhythmias and heart failure. Moreover, the association between type 2 diabetes and heart disease remains statistically significant, even after fully controlling for genetic and early-life familial environmental background. Patients with type 2 diabetes who reported maintaining a healthy lifestyle consisting of being a non-smoker, no/mild alcohol consumption, regular physical activity and being non-overweight had a significantly lower heart disease risk than those with an unfavourable lifestyle. Our findings highlight the importance of a healthy lifestyle in prevention of heart disease among patients with type 2 diabetes.

## Supplementary Information

ESM (PDF 140 kb)

## Data Availability

Raw data are available by request from qualified investigators applying to the Swedish Twin Registry.

## Abbreviations

AP: Attributable proportion
GEE: Generalised estimating equation
NPR: National Patient Registry
RERI: Relative excess risk due to interaction
SALT: Screening Across the Lifespan Twin study
SI: Synergy index
STR: Swedish Twin Registry
