# Supplementary material for: A healthy lifestyle mitigates the risk of heart disease related to type 2 diabetes: a prospective nested case–control study in a nationwide Swedish twin cohort
Source: Diabetologia. 2020 Nov 10;64(3):530–9. doi: 10.1007/s00125-020-05324-z (PMC7864843; doi:10.1007/s00125-020-05324-z)
Supplement: Supplementary file 1 — (PDF 140 kb) [file 125_2020_5324_MOESM1_ESM.pdf]

Electronic supplementary material (ESM)

ESM Table 1. Odds ratios (ORs) and 95% confidence intervals (CIs) of heart disease in relation to lifestyle among people with and without type 2 diabetes from Generalized Estimating Equation models (unfavourable lifestyle as reference)

ESM Table 2. Additive interaction between lifestyle and type 2 diabetes (T2D) for the risk of heart disease.

ESM Table 3. Odds ratios (ORs) and 95% confidence intervals (CIs) of different forms of heart disease related to type 2 diabetes from Generalized Estimating Equation models (type 2 diabetes-free as the reference) among men and women

ESM Table 4. Odds ratios (ORs) and 95% confidence intervals (CIs) of different forms of heart disease related to type 2 diabetes (type 2 diabetes-free as the reference) from Generalized Estimating Equation models further adjusted for survival status.

ESM Table 5. Odds ratios (ORs) and 95% confidence intervals (CIs) of different forms of heart disease related to type 2 diabetes (type 2 diabetes-free as the reference) from Generalized Estimating Equation models using data available (excluding data with missing values for covariate) (n=35,445)

ESM Table 1. Odds ratios (ORs) and 95% confidence intervals (CIs) of heart disease in relation to lifestyle among people with and without type 2 diabetes from Generalized Estimating Equation models (unfavourable lifestyle as reference)

Lifestyle	type 2 diabetes				type 2 diabetes-free			
	Subjects	Cases	Basic-adjusted OR (95% CI)*	Multi-adjusted OR (95% CI)†	Subjects	Cases	Basic-adjusted OR (95% CI)*	Multi-adjusted OR (95% CI)†
Unfavourable	211	143	Reference	Reference	2404	550	Reference	Reference
Intermediate	1713	1060	0.76 (0.56, 1.03)	0.68 (0.49, 0.93)	26830	5578	0.87 (0.79, 0.96)	0.74 (0.66, 0.82)
Favourable	380	201	0.52 (0.37, 0.74)	0.44 (0.30, 0.63)	9925	1730	0.70 (0.63, 0.78)	0.55 (0.49, 0.62)

***** Unadjusted.

† Adjusted for age, sex, education, and marital status.

ESM Table 2. Additive interaction between lifestyle and type 2 diabetes (T2D) for the risk of heart disease

Joint exposure		No. of subjects	Heart disease		
Lifestyle	T2D		Cases	Basic-adjusted OR (95% CI)*	Multi-adjusted OR (95% CI)†
Intermediate/ Favourable	No	36,755	7308	Reference	Reference
Unfavourable	No	2404	550	1.21 (1.10, 1.34)	1.42 (1.28, 1.57)
Intermediate/ Favourable	Yes	2093	1261	5.87 (5.35, 6.43)	4.54 (4.10, 5.04)
Unfavourable	Yes	211	143	8.39 (6.22, 11.31)	8.47 (6.14 11.69)

***** Unadjusted.

† Adjusted for age, sex, education, marital status.

Measures of additive interaction for heart disease:

Relative excess risk due to interaction (RERI): 3.507, 95% CI: 0.929, 6.084

Attributable proportion due to interaction (AP): 0.414, 95% CI: 0.231, 0.597

Synergy index (SI): 1.885, 95% CI: 1.318, 2.696

ESM Table 3. Odds ratios (ORs) and 95% confidence intervals (CIs) of different forms of heart disease related to type 2 diabetes from Generalized Estimating Equation models (type 2 diabetes-free as the reference) among men and women

	Males			Females		
Heart Disease	Cases	OR (95% CI)*	OR (95% CI)†	Cases	OR (95% CI)*	OR (95% CI)†
All types heart Disease	4955	4.45 (3.87, 5.10)	4.10 (3.57, 4.70)	4307	5.06 (4.40, 5.82)	4.71 (4.09, 5.43)
Coronary heart disease	2568	4.48 (3.82, 5.25)	4.20 (3.58, 4.93)	1835	6.10 (5.17, 7.20)	5.75 (4.86, 6.80)
Angina pectoris	1060	4.01 (3.24, 4.96)	3.79 (3.07, 4.69)	876	5.28 (4.23, 6.59)	4.91 (3.92, 6.15)
Acute myocardial infarction	1296	4.32 (3.55, 5.26)	4.12 (3.38, 5.02)	793	6.58 (5.31, 8.17)	6.31 (5.06, 7.86)
Chronic ischemic heart disease	205	5.13 (3.47, 7.60)	4.70 (3.16, 6.99)	157	6.01 (3.86, 9.36)	5.86 (3.74, 9.16)
Other coronary heart disease	7	20.70 (4.50, 95.22)	-	9	10.99 (1.89, 63.74)	-
Cardiac arrhythmias	1750	3.42 (2.83, 4.13)	3.24 (2.69, 3.91)	1721	3.30 (2.71, 4.02)	3.07 (2.52, 3.74)
Atrial fibrillation and flutter	1484	3.47 (2.84, 4.23)	3.27 (2.68, 3.99)	1351	3.35 (2.70, 4.17)	3.04 (2.45, 3.78)
Other cardiac arrhythmias	266	3.03 (1.99, 4.63)	3.04 (1.99, 4.65)	370	3.02 (2.05, 4.44)	3.05 (2.07, 4.50)
Heart failure	637	5.13 (3.98, 6.62)	4.61 (3.57, 5.96)	751	5.51 (4.31, 7.05)	5.16 (4.02, 6.62)
Congestive heart failure	87	7.59 (4.54, 12.69)	6.92 (4.19, 11.41)	94	5.17 (2.92, 9.16)	4.57 (2.57, 8.15)
Left ventricular failure	48	5.07 (2.40, 10.73)	4.93 (2.35, 10.37)	62	4.80 (2.32, 9.92)	4.12 (2.02, 8.44)
Unspecified heart failure	502	4.47 (3.37, 5.92)	3.98 (2.99, 5.30)	595	5.29 (4.06, 6.89)	5.10 (3.89, 6.68)

***** Adjusted for age and education.

† Adjusted for age, education, marital status, body mass index, smoking, alcohol consumption, and physical activity.

ESM Table 4. Odds ratios (ORs) and 95% confidence intervals (CIs) of different forms of heart disease related to type 2 diabetes (type 2 diabetes-free as the reference) from Generalized Estimating Equation models further adjusted for survival status.

Heart Disease	No. of Cases	OR (95% CI)*
All types heart Disease	9262	4.30 (3.90, 4.75)
Coronary heart disease	4403	4.84 (4.31, 5.45)
Angina pectoris	1936	4.39 (3.75, 5.13)
Acute myocardial infarction	2089	4.81 (4.14, 5.58)
Chronic ischemic heart disease	362	5.13 (3.81, 6.91)
Other coronary heart disease	16	14.73 (4.34, 49.96)
Cardiac arrhythmias	3471	3.14 (2.74, 3.60)
Atrial fibrillation and flutter	2835	3.12 (2.69, 3.61)
Other cardiac arrhythmias	636	3.20 (2.40, 4.27)
Heart failure	1388	4.44 (3.70, 5.33)
Congestive heart failure	181	4.86 (3.35, 7.06)
Left ventricular failure	110	3.90 (2.33, 6.53)
Unspecified heart failure	1097	4.16 (3.41, 5.08)

***** Adjusted for age, sex, education, marital status, body mass index, smoking, alcohol consumption, physical activity, and death.

ESM Table 5. Odds ratios (ORs) and 95% confidence intervals (CIs) of different forms of heart disease related to type 2 diabetes (type 2 diabetes-free as the reference) from Generalized Estimating Equation models using data available (excluding data with missing values for covariate) (n=35,445)

Heart Disease	No. of Cases	OR (95% CI)*
All types heart Disease	6654	4.85 (4.32, 5.43)
Coronary heart disease	3374	
Angina pectoris	1567	4.40 (3.67, 5.27)
Acute myocardial infarction	1513	5.35 (4.50, 6.37)
Chronic ischemic heart disease	279	5.07 (3.56, 7.23)
Other coronary heart disease	15	16.80 (5.13, 54.98)
Cardiac arrhythmias	2554	
Atrial fibrillation and flutter	1992	3.66 (3.09, 4.33)
Other cardiac arrhythmias	562	3.29 (2.42, 4.47)
Heart failure	726	
Congestive heart failure	95	9.58 (5.96, 15.37)
Left ventricular failure	62	7.10 (3.72, 13.56)
Unspecified heart failure	569	5.92 (4.65, 7.54)

***** Adjusted for age, sex, education, marital status, body mass index, smoking, alcohol consumption, and physical activity.
